# First karyotype description and nuclear 2C value for *Myrsine* (Primulaceae): comparing three species

**DOI:** 10.3897/CompCytogen.v11i1.11601

**Published:** 2017-03-13

**Authors:** Renata Flávia de Carvalho, Paulo Marcos Amaral-Silva, Micheli Sossai Spadeto, Andrei Caíque Pires Nunes, Tatiana Tavares Carrijo, Carlos Roberto Carvalho, Wellington Ronildo Clarindo

**Affiliations:** 1 Laboratório de Citogenética, Departamento de Biologia, Centro de Ciências Agrárias, Universidade Federal do Espírito Santo, 29.500-000 Alegre (ES), Brazil; 2 Laboratório de Biometria, Departamento de Biologia Geral, Universidade Federal de Viçosa, 36.570-000 Viçosa (MG), Brazil; 3 Laboratório de Botânica, Departamento de Biologia, Centro de Ciências Agrárias, Universidade Federal do Espírito Santo, 29.500-000 Alegre (ES), Brazil; 4 Laboratório de Citogenética e Citometria, Departamento de Biologia Geral, Centro de Ciências Biológicas e da Saúde, Universidade Federal de Viçosa, 36.570-000 Viçosa (MG), Brazil

**Keywords:** Atlantic Forest, cytogenetics, flow cytometry, karyogram, Myrsinaceae, *
Rapanea
*

## Abstract

Cytogenetic studies in Primulaceae are mostly available for herbaceous species, and are focused on the chromosome number determination. An accurate karyotype characterization represents a starting point to know the morphometry and class of the chromosomes. Comparison among species within *Myrsine*, associating these data with the nuclear 2C value, can show changes that led the karyotype evolution. Here, we studied three *Myrsine* species [*Myrsine coriacea* (Swartz, 1788) Brown ex Roemer et Schultes, 1819, *Myrsine umbellata* Martius, 1841 and *Myrsine parvifolia* Candolle, 1841] that show different abilities to occupy the varied types of vegetation within the Brazilian Atlantic Forest. Cytogenetic characterization showed some individuals with 2n = 45 chromosomes for *Myrsine parvifolia* and *Myrsine coriacea*, with most individuals of the three species having 2n = 46. The first karyograms for *Myrsine* were assembled and presented morphologically identical and distinct chromosome pairs. In addition, differences in the mean 2C nuclear value and chromosome morphometry were found. Therefore, the first description of the *Myrsine* karyotype has been presented, as well as the nuclear 2C value. The procedures can be applied to other *Myrsine* species for future investigations in order to better understand its effects on the differential spatial occupation abilities shown by the species in Brazilian Atlantic Forest.

## Introduction

Previous studies regarding the chromosome number in Primulaceae (s. APG 2016) are available for some genera, as: *Cyclamen* Linnaeus, 1753 (Bennett and Grimshaw 1991, Ishizaka 2003), *Anagallis* Linnaeus, 1753 (Aguilera et al. 2011, Bennett and Leitch 2012), *Lysimachia* Linnaeus, 1753 (Baltisberger and Kocyan 2010, Bennett and Leitch 2012, Chalup and Seijo 2013), *Androsace* Linnaeus, 1753 (Chepinoga et al. 2009), *Elingamita* Baylis, 1951 (Dawson 1995), *Trientalis* Linnaeus, 1753 (Vickery and Miller 2008), *Ardisia* Swartz, 1788 (Koyama and Kokubugata 1998), *Primula* Linnaeus, 1753 (Abou-El-Enain 2006, Casazza et al. 2012, Theodoridis et al. 2013), and *Dodecatheon* Linnaeus, 1753 (Oberle et al. 2012), and *Myrsine* Linnaeus, 1753 (Beuzenberg and Hair 1983, Dawson 1995, 2000, Hanson et al. 2003, Rice et al. 2015). Except the genus *Cyclamen* and *Myrsine*, these taxa comprise annual and biennial herbaceous species.

The cosmopolitan *Myrsine* Linnaeus is one of the main genera of Primulaceae, considering species richness, represented by tree and shrub species (Heenan and Lange 1998). Its members are generally dioecious plants, characterized by ramiflorus and congested inflorescences, and flowers with oppositipetalous stamens. Despite *Myrsine* being one of the largest and most important genera of Primulaceae, only eighteen species, among the 300 estimated from this genus, have been studied regarding cytogenetic aspects. Fifteen of these species occur in the African, Asian and Oceania continents (*Myrsine coxii* Cochayne, 1902, *Myrsine divaricata* Cunningham, 1839, *Myrsine kermadecensis* Cheeseman, 1887, *Myrsine nummularia* (Hooker f.) Hooker f., 1867, *Myrsine salicina* (Hooker f.) Hooker f., 1864, *Myrsine argentea* Heenan et de Lange, 1998, *Myrsine oliveri* Allan, 1961, *Myrsine chathamica* Mueller, 1864; *Myrsine africana* Linnaeus, 1753; *Myrsine sandwicensis* Candolle, 1841, *Myrsine seguinii* Léveille, 1914, *Myrsine semiserrata* Wallich, 1824, *Myrsine australis* (A. Richard, 1832) Allan, 1947, *Myrsine capitellata* Wallich, 1824), and just three occurs in America continent (*Myrsine matensis* (Mez, 1902) Otegui, 1998; *Myrsine guianensis* (Aublet, 1775) Kuntze, 1891, *Myrsine coriacea* (Swartz, 1788) Brown ex Roemer et Schultes, 1819. The chromosome number (2n = 46 or 2n = 48) was the only karyotype data reported, without any images of the chromosomes. In addition, the evolutionary aspects that culminated in the karyotype diversification within the genus are poorly understood.

One interesting ecological aspect observed in Neotropical species of *Myrsine* that occur in Brazil is that some of them occur in more than one biome, as Cerrado, Atlantic Forest, and Amazonian Forest, while others are restricted of one of these biomes, as Atlantic Forest (BFG 2015). Among species that occur in Atlantic Forest, for example, some are able to occupy different types of vegetation within this biome, including Restinga Vegetation, High Altitude Campos, Rocky Outcrops, Ombrophyllous and Mixed Ombrophyllous Forests, while others are able to occupy just one type of vegetation (Freitas and Kinoshita 2015). Considering the distinct ecological aspects, cytogenetic studies are relevant to show other differences between these species.

Studies combining cytogenetics and nuclear DNA content have offered data for understanding evolutionary processes in different species (Clarindo and Carvalho 2008, Kolář et al. 2013). Measurement of the nuclear DNA content is complementary to cytogenetic information and is useful for detecting genome size variations between related species (Marhold et al. 2010, Kolář et al. 2013). Fine adjustments in cytogenetic procedures, combining advances in microscopy and image analysis systems, can provide accurate karyotype characterization for *Myrsine* species. Here, we study three species of *Myrsine* that occur in contrasting types of vegetation of the Brazilian Atlantic Forest, aiming to determine the chromosome number, describe the karyotype and measure the nuclear DNA.

## Material and methods

### Plant samples

Three species were selected for this study: 1. *Myrsine coriacea* (Voucher – T.T. Carrijo 1458, VIES herbarium), which is a widespread species in Atlantic Forest found in all types of vegetation, including open areas within Ombrophyllous and Mixed Ombrophyllous Forests, Rock Outcrops, High Altitude Campos, and Restinga Vegetation; 2. *Myrsine umbellata* (Voucher – T.T. Carrijo 1467, VIES herbarium), which is found in mostly all types of vegetation of *Myrsine coriacea*, except High Altitude Campos; and 3. *Myrsine parvifolia* (Voucher – T.T. Carrijo 2232, VIES herbarium), a species restricted to Restinga vegetation (BFG 2015).

Fruits and leaves of all species were collected. *Myrsine coriacea* and *Myrsine umbellata* were sampled from October 2012 to July 2015 in a forest remnant located in Iúna municipality, Espírito Santo (ES) State, Brazil (20°21'6"S – 41°31'58"W), characterized as Rocky Outcrops, at 600 (*Myrsine coriacea*) and 1,100 m.s.m (*Myrsine umbellata*). *Myrsine parvifolia* was collected in a forest remnant located in Guarapari municipality, ES, Brazil (20°36'15"S – 40°25'27"W), characterized as coastal sandy plains vegetation (Restinga) at sea level. Leaves and fruits of *Solanum lycopersicum* L. and *Pisum sativum* L. (internal standards for flow cytometry – FCM, 2C = 2.00 pg and 2C = 9.16 pg, respectively, Praça-Fontes et al. 2011) were supplied by Dr. Jaroslav Doležel (Experimental Institute of Botany, Czech Republic).

### In vitro plantlet recovering

Fruit pericarp was manually removed and the seeds were desinfested according to Oliveira et al. (2013) and germinated in a medium composed of MS salts (Sigma) and vitamins (Murashige and Skoog 1962), 30 g L^-1^ sucrose (Sigma), 7 g L^-1^ agar and 2.685 µM naphthaleneacetic acid (NAA, Sigma). *Solanum lycopersicum* and *Pisum sativum* seeds were subjected to the same disinfestation procedure and inoculated in medium without NAA. Germination was done at 25 °C under a 16/8 hours (light/dark) regime.

### Nuclear 2C value measurement

In order to adapt the FCM for *Myrsine*, the following procedures were done: (a) initially, from leaves collected in the field of male and female individuals (samples) and of the two standards; (b) afterward, replacing the dithiothreitol antioxidant by polyethylene glycol (PEG) in nuclei isolation buffer; and (c) from leaves of the samples and *Pisum sativum* plantlets in vitro cultivated.

Nuclei suspensions were obtained by co-chopping (Galbraith et al. 1983) leaf fragments (1 cm^2^) cut from each sample (*Myrsine* species) and standard (*Solanum lycopersicum* or *Pisum sativum*). The suspensions were processed and stained following Otto (1990) and Praça-Fontes et al. (2011) and analyzed with the flow cytometer Partec PAS II/III (Partec GmbH). *Myrsine* genome size was measured by multiplying the 2C value of the internal standard using the fluorescence intensity corresponding to G_0_/G_1_ nuclei peak. Mean 2C values were compared by the *F* test at 5% probability.

### Cytogenetic analysis

Roots were cut from the in vitro plantlets, treated with 5.0 μM amiprofos-methyl (APM) (Agrochem KK Nihon Bayer) for 12, 15, 18 or 24 h at 4°C, rinsed in distilled water (dH_2_O) for 20 min and fixed in methanol:acetic acid (3:1) for 24 h. The fixative solution was changed three times and the material was stored at -20°C (Carvalho et al. 2007). The roots were washed, macerated in 1:5 pectinase solution (enzyme:dH_2_O) for 3 h at 34°C, or 1:20 enzymatic pool (4% cellulase – Kinki Yakult MFG, 1% macerozyme – Kinki Yakult MFG, and 0.4% hemicellulase – Sigma) for 1 h 30 min or 1 h 45 min at 34°C, washed in dH_2_O, fixed, and stored at -20°C.

Slides were prepared and stained according to Carvalho et al. (2007) and analyzed on a Nikon eclipse Ci-S microscope (Nikon). Prometaphases and metaphases were captured using the 100× objective and a CCD camera (Nikon Evolution^TM^) coupled to a Nikon microscope 80i (Nikon). About 100 slides were analyzed for each *Myrsine* species. Chromosome morphometry was characterized and the class was determined as proposed by Levan et al. (1964) and reviewed by Guerra (1986).

Using chromosome morphometric data (total, short and long arm length), the standardized Euclidean Distance and Unweighted Pair-Group Method Average (UPGMA) was applied to each species. In addition, the value of the relative size (% size in relation to sum of the mean values of total length, Table 1) of each chromosome was compared among species by the Scott-Knott test at 5% probability. Analyses were made using the software R 3.2.4 (R Core Team 2016).

**Table 1.** Morphometry and chromosome class performed at least 10 prometaphases/metaphases. In all species were found chromosomes morphologically indentical, similar and distinct.

* Myrsine parvifolia *							* Myrsine coriacea *						* Myrsine umbellata *					
Chrom.	Total ± SD	Short	Long	r	Class	Relative size (%)	Total ± SD	Short	Long	r	Class	Relative size (%)	Total ± SD	Short	Long	r	Class	Relative size (%)
1	2.64 ± 0.29	1.01	1.63	1.61	SM	5.60	2.79 ± 0.09	1.24	1.55	1.25	M	6.14	2.72 ± 0.06	1.14	1.59	1.39	M	6.60
2	2.47 ± 0.23	1.09	1.37	1.25	M	5.24	2.45 ± 0.11	1.02	1.42	1.38	M	5.38	2.67 ± 0.06	1.14	1.54	1.35	M	6.48
3	2.45 ± 0.22	0.86	1.59	1.85	SM	5.19	2.35 ± 0.10	1.09	1.26	1.15	M	5.17	2.13 ± 0.16	0.94	1.19	1.26	M	5.16
4	2.44 ± 0.27	0.68	1.75	2.55	SM	5.17	2.30 ± 0.05	1.02	1.27	1.24	M	5.06	2.13 ± 0.08	0.84	1.29	1.53	SM	5.16
5	2.24 ± 0.18	0.71	1.53	2.13	SM	4.76	2.29 ± 0.08	0.99	1.30	1.30	M	5.04	2.08 ± 0.14	0.74	1.34	1.80	SM	5.04
6	2.21 ± 0.17	0.73	1.48	2.00	SM	4.69	2.22 ± 0.17	0.86	1.36	1.57	SM	4.88	1.88 ± 0.11	0.64	1.24	1.92	SM	4.56
7	2.18 ± 0.25	0.81	1.37	1.68	SM	4.62	2.17 ± 0.12	0.78	1.39	1.77	SM	4.77	1.83 ± 0.11	0.79	1.04	1.31	M	4.44
8	2.16 ± 0.27	0.61	1.55	2.51	SM	4.59	2.12 ± 0.11	0.78	1.34	1.71	SM	4.67	1.83 ± 0.09	0.59	1.24	2.08	SM	4.44
9	2.15 ± 0.29	0.86	1.29	1.49	M	4.55	2.04 ± 0.15	0.81	1.23	1.50	SM	4.49	1.83 ± 0.09	0.59	1.24	2.08	SM	4.44
10	2.13 ± 0.25	0.61	1.51	2.45	SM	4.51	2.00 ± 0.10	0.78	1.23	1.56	SM	4.41	1.78 ± 0.12	0.59	1.19	2.00	SM	4.32
11	2.09 ± 0.22	0.79	1.31	1.65	SM	4.44	2.00 ± 0.17	0.75	1.26	1.67	SM	4.41	1.68 ± 0.13	0.59	1.09	1.83	SM	4.08
12	1.99 ± 0.16	0.75	1.23	1.63	SM	4.22	1.89 ± 0.10	0.71	1.18	1.64	SM	4.16	1.68 ± 0.08	0.59	1.09	1.83	SM	4.08
13	1.97 ± 0.23	0.66	1.31	1.96	SM	4.19	1.89 ± 0.07	0.57	1.32	2.31	SM	4.16	1.68 ± 0.10	0.49	1.19	2.40	SM	4.08
14	1.95 ± 0.14	0.65	1.30	2.00	SM	4.14	1.84 ± 0.06	0.55	1.29	2.32	SM	4.06	1.68 ± 0.14	0.66	1.02	1.52	SM	4.08
15	1.93 ± 0.16	0.72	1.21	1.67	SM	4.11	1.81 ± 0.11	0.65	1.16	1.78	SM	3.98	1.58 ± 0.06	0.64	0.94	1.46	M	3.84
16	1.85 ± 0.13	0.65	1.20	1.82	SM	3.93	1.81 ± 0.08	0.57	1.24	2.17	SM	3.98	1.58 ± 0.06	0.69	0.89	1.29	M	3.84
17	1.85 ± 0.23	0.72	1.13	1.56	SM	3.93	1.78 ± 0.04	0.66	1.11	1.66	SM	3.91	1.58 ± 0.09	0.69	0.89	1.29	M	3.84
18	1.84 ± 0.19	0.70	1.15	1.63	SM	3.92	1.71 ± 0.13	0.65	1.06	1.63	SM	3.77	1.58 ± 0.11	0.59	0.99	1.67	SM	3.84
19	1.82 ± 0.22	0.63	1.20	1.89	SM	3.87	1.68 ± 0.11	0.55	1.13	2.03	SM	3.70	1.58 ± 0.08	0.59	0.99	1.67	SM	3.84
20	1.75 ± 0.18	0.68	1.06	1.55	SM	3.71	1.67 ± 0.09	0.58	1.09	1.85	SM	3.69	1.58 ± 0.13	0.49	1.09	2.20	SM	3.84
21	1.68 ± 0.14	0.79	0.89	1.13	M	3.56	1.55 ± 0.16	0.49	1.06	2.17	SM	3.42	1.43 ± 0.14	0.59	0.84	1.42	M	3.48
22	1.66 ± 0.16	0.58	1.08	1.85	SM	3.53	1.55 ± 0.04	0.35	1.20	3.33	A	3.42	1.38 ± 0.11	0.49	0.89	1.80	SM	3.36
23	1.66 ± 0.30	0.58	1.08	1.86	SM	3.53	1.52 ± 0.07	0.39	1.13	2.88	SM	3.34	1.28 ± 0.10	0.59	0.69	1.17	M	3.13
Sum	47.22	16.99	30.23			100.00	45.53	16.96	28.57			100.00	41.30	15.79	25.51			100.00

Chrom = chromosomes; Total = total length; SD = standard deviation; Long/Short = arm length; r = arm ratio – long/short; M = metacentric; SM = submetacentric; A = acrocentric; Relative size = % size in relation to sum of the mean values of total length; Sum = sum of the mean values.

## Results

### In vitro plantlet recovering

Approximately 60 days after in vitro inoculation, plantlets were obtained for the three *Myrsine* species. All plantlets exhibited sufficient and morphologically normal leaves and roots for FCM and cytogenetic analyses, respectively.

### Nuclear 2C value measurement

FCM analysis performed on leaves collected in the field did not result in histograms showing profile G_0_/G_1_ peaks. So, dithiothreitol antioxidant was replaced by PEG in the nuclei isolation buffer OTTO I. This change provided G_0_/G_1_ peaks, exhibiting a coefficient of variation (CV) less than 5% for *Myrsine umbellata* and the two internal standards. The channel of the *Pisum sativum* G_0_/G_1_ peak however was closer to *Myrsine umbellata* than *Solanum lycopersicum* Thus, based on linearity international criteria for FCM, *Pisum sativum* was the standard chosen for the next measurements. The mean 2C value of the male (2C = 6.65 pg ± 0.02) and female (2C = 6.67 pg ± 0.11) *Myrsine umbellata* individuals were statistically identical by the *F* test. Considering these previous results, the 2C value was measured from leaves of in vitro plantlets. The mean values were 2C = 4.81 pg ± 0.05 for *Myrsine parvifolia*, 2C = 6.60 pg ± 0.14 for *Myrsine coriacea* and 2C = 6.63 pg ± 0.13 for *Myrsine umbellata*. The mean value of the *Myrsine umbellata* in vitro plantlets was statistically identical to the males and females in the field. Therefore, the mean value adopted for this species was 2C = 6.65 pg, which was statistically equal to the *Myrsine coriacea*.

### Cytogenetic analysis

**Figure 1.**
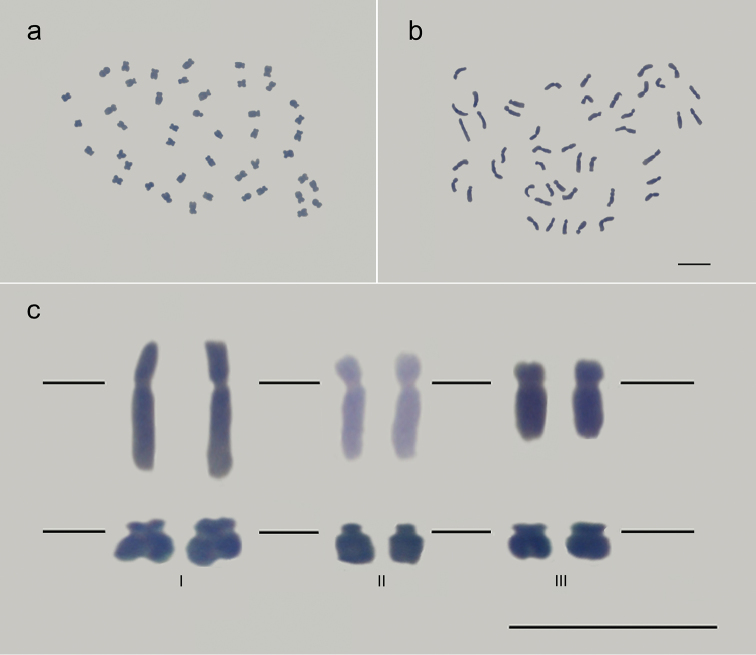
First images of the *Myrsine* chromosomes. Karyotype of a *Myrsine parvifolia* individual with 2n = 45 (**a**) and another with 2n = 46 (**b**) chromosomes. Note the different levels of chromatin compaction between the chromosomes of the two karyotypes. The distinct chromatin compact level was highlighted in (**c**), where the same submetacentric chromosome of *Myrsine parvifolia* (above) and the same acrocentric chromosome of *Myrsine coriacea* (below) were taken from two different prometaphases (I and II) and one metaphase (III). Bar = 5 µm.

Roots exposed to a 12 h APM provided prometaphases, exhibiting chromosomes at a distinct chromatin compact level, and metaphases. Enzymatic maceration in 1:5 pectinase solution ensured the chromosomes remained inside the cell, allowing an accurate determination of 2n = 45 or 2n = 46. Chromosome number of 2n = 45 was found for 12.60% individuals of *Myrsine parvifolia* and 8.45% of *Myrsine coriacea*, with 2n = 46 for the three species. Based on these results, the next slides were made from roots of particular seedlings with 2n = 45 or 2n = 46. Root maceration with 1:20 enzymatic pool for 1h 30 min supplied chromosomes no damage to the chromatin structure, without overlapping, with well-defined centromeres and free of cytoplasm debris (Fig. 1).

**Figure 2.**
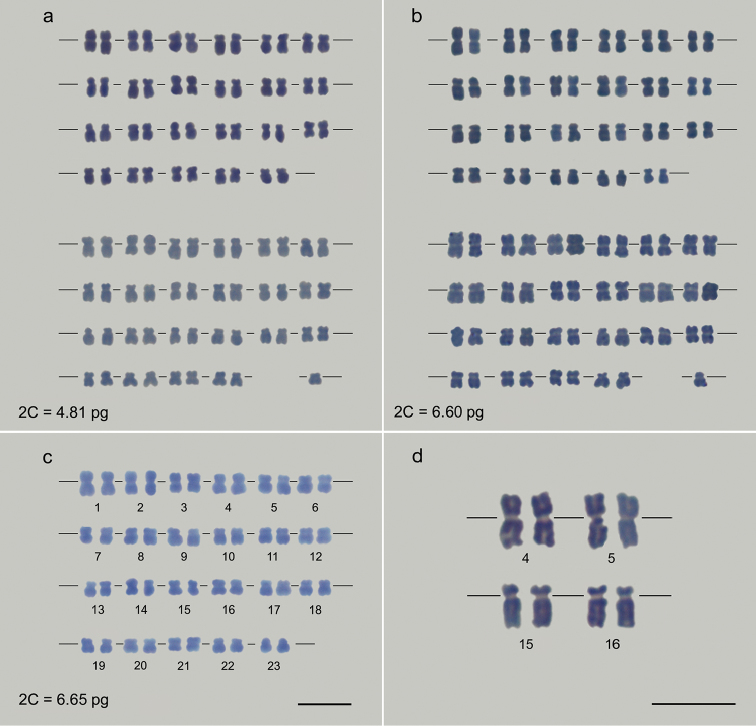
*Myrsine* karyograms displaying 2n = 45 (**a** *Myrsine parvifolia* and **b** *Myrsine coriacea*) or 2n = 46 chromosomes (**a**–**c** the three species). In all *Myrsine parvifolia* (**a**) and *Myrsine coriacea* (**b**) individuals with 2n = 45, the odd chromosome number was well-marked by absence of the homologue pair of the chromosome 23. Metacentric and submetacentric chromosomes prevailing in the karyograms of the three species, with only one acrocentric chromosome was identified in *Myrsine coriacea* (**b** chromosome 22). Although showing approximately 2C = 1.50 pg less DNA, *Myrsine parvifolia* (a) displayed the same chromosome number in relation to the other species (**b** *Myrsine coriacea* **c** *Myrsine umbellata*). For all species, morphometric analyses showed identical, similar and distinct chromosome pairs with regard to morphometry and class. The similarity of some chromosomes was highlighted from the metacentric chromosome pairs 4 and 5 (**d** above) and submetacentric 15 and 16 (**d** below) of *Myrsine coriacea*. In contrast, other chromosomes showed singular morphology, as the chromosome 1 and 2 of all species, the 22 of *Myrsine coriacea*, which is the single acrocentric chromosome, and the chromosome 23. Bar = 5 µm.

Karyotype characterization was possible only after carefully testing the time and concentration of the APM antitubulin and cell wall enzymes. *Myrsine parvifolia* presented a greater total sum of the length of the chromosomes despite having less nuclear DNA content. For this species only, we found prometaphase chromosomes showing low level of chromatin compaction (Fig. 2a), resulting in a higher sum of the total length (Table 1). *Myrsine coriacea* and *Myrsine umbellata* did not show pronounced variation in chromatin compaction, but the quality of the chromosomes allowed us to characterize the karyotype and to assemble the karyogram (Fig. 2b–c, Table 1).

Morphometric analysis was used to classify the chromosomes and evidence similarities and differences among species karyotypes. *Myrsine parvifolia* presented three metacentric (2, 9 and 21) and 20 submetacentric (1, 3–8, 10–20, 22 and 23) chromosome pairs, *Myrsine coriacea* showed five metacentric (1–5), 17 submetacentric (6–21 and 23) and one acrocentric (22) chromosome pairs, and *Myrsine umbellata* displayed nine metacentric (1–3, 7, 15–17, 21 and 23) and 14 submetacentric (4–6, 8–14, 17, 18, 20 and 22) chromosome pairs (Fig. 2, Table 1).

**Figure 3.**
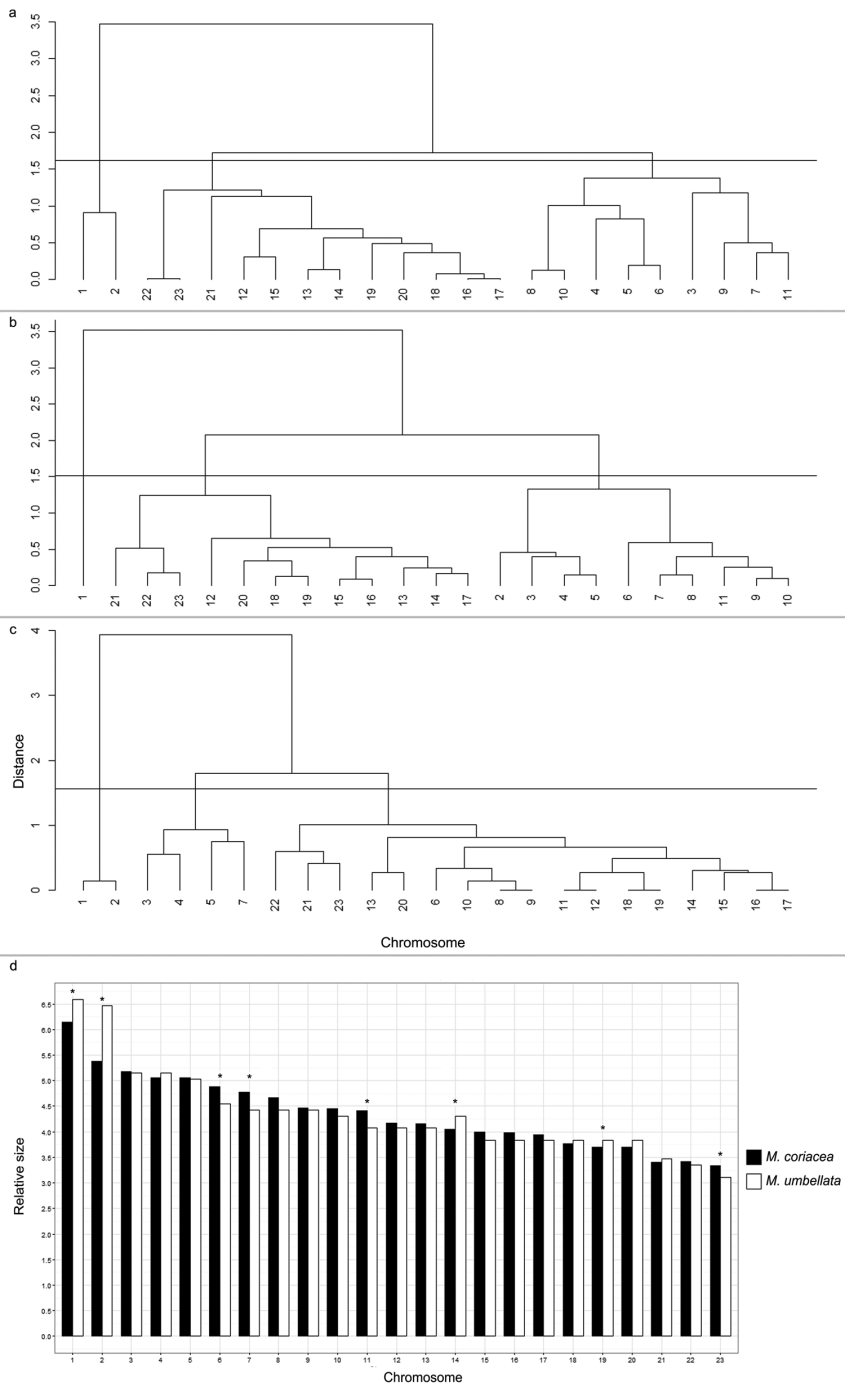
**a–c** Multivariate clustering generated from chromosome morphometric data (total, long and short arms length). Mojena’s criteria showed three clusters for *Myrsine parvifolia* (**a**), *Myrsine coriacea* (**b**) and *Myrsine umbellata* (**c**) with cut point between 1.5 to 1.8. This analysis confirmed the morphological discrepancy of the chromosome 1, and the similarity of other chromosomes (**d**) Graphic provided by comparison between mean relative size (% size in relation to sum of the mean values of total length, Table 1) of each chromosome of *Myrsine coriacea* and *Myrsine umbellata*. The chromosomes 1, 2, 6, 7, 11, 14, 19 and 23 (*) between the species are statistically different in relation to mean relative size according to Scott Knott test at 5% of probability.

Morphologically similar and identical chromosomes groups were found in all species. *Myrsine parvifolia* presented sets of two chromosome pairs (5–6, 13–14, 16–17 and 22–23), as did *Myrsine coriacea* (4–5, 10–11, 13–14, 15–16 and 19–20), and *Myrsine umbellata* presented three sets of two (11–12, 16–17 and 18–19) and one set of three chromosome pairs (8–10). The other chromosome pairs in each species were considered morphologically distinct (Fig. 2, Table 1, 2). Using morphometric data and applying the UPGMA statistical analysis, the chromosomes of each *Myrsine* species were grouped in three clusters in all species (Fig. 3a–c, Table 2). Chromosome groups formed by qualitative analysis of all species were clustered by UPGMA, supporting previous findings.

As the mean 2C values of *Myrsine coriacea* (6.60 pg) and *Myrsine umbellata* (6.65 pg) were statistically identical, the Scott-Knott test was used to compare the relative size (Table 1) of each chromosome of these species. Chromosomes 1, 2, 6, 7, 11, 14, 19 and 23 differed between the species, while the others were statistically identical (Fig. 3d, Table 2).

**Table 2.** Chromosome groups of the *Myrsine* karyotype suggested from karyogram evaluation (Fig. 2 and Table 1) and confirmed by UPGMA clustering (Fig. 3a–c).

Species	*Karyogram evaluation	**UPGMA clustering	***Confirmed chromosome groups
* Myrsine parvifolia *	5–6; 13–14; 16–17; and 22–23	1 and 2; 3–11; and 12–23	5–6; 13–14; 16–17; and 22–23
* Myrsine coriacea *	4–5; 9–10; 13–14; 15–16; and 19–20	1; 2–11; and 12–23	4–5; 9–10; 13–14; 15–16; and 19–20
* Myrsine umbellata *	8–10; 11–12; 16–17; and 18–19	1 and 2; 3–5, 7; and 6, 8–23	8–10; 11–12; 16–17; and 18–19

* Chromosome groups morphologically identical or similar defined from all morphometric data (total length, short and long arms, r = ratio long/short arm, chromosomal class; relative size) and observation of the karyogram. ** Chromosome groups formed by UPGMA clustering method using data about total, short and long arms length. *** Common chromosome groups evidenced by two analyses (qualitative *x* quantitative).

## Discussion

The first step in FCM was to define the best antioxidant and internal standard. The presence of secondary metabolites in the *Myrsine* leaves, such as tannins, saponins, flavonoids and steroids (Abbi et al. 2011) made this challenging. These compounds probably prevented us from measuring the 2C value in individuals from the field when the OTTO I buffer (Otto 1990) was supplemented with dithiothreitol. Cytosolic compounds can reduce or inhibit the interaction of the fluorochromes and DNA during the nuclei staining step (Noirot et al. 2003). Antioxidants inhibit this interference, preserving the chromatin structure (Shapiro 2003). Nevertheless, the dithiothreitol was not efficient at providing nuclei suspensions suitable for FCM. Thus, this compound, which is more specific for molecules that possess free sulfhydryl groups, was replaced by PEG because of its wide spectrum for antioxidant activities, an effect called PEGylation (Term Fisher Scientific 2016). Due to this effect, PEG was more efficient at inhibiting the action of cytosolic compounds, resulting in G_0_/G_1_ peaks for *Myrsine umbellata* and *Pisum sativum* with CV below 5%. Owing to the linearity parameter, *Pisum sativum* was a more adequate standard relative to *Solanum lycopersicum*, which reduced measurement errors.

Secondary metabolite interference was completely resolved for other *Myrsine* species using in vitro plantlets propagated in a controlled environment. FCM measurements from leaves collected in the field may have been influenced by environmental conditions. Secondary metabolite production is influenced by humidity, temperature, light intensity and the availability of water and nutrients (Akula and Ravishankar 2011). Thus, the conditions at each elevation gradient can be associated with the FCM result, suggesting a differentiated production of secondary metabolic compounds for *Myrsine* at distinct altitudes.

Genome size in *Myrsine* had only been reported for *Myrsine africana* as 2C = 2.46 pg (Hanson et al. 2003), which was measured by Feulgen microdensitometry using *Vigna* sp. as standard. Levels of endoreduplication in cells of *Vigna radiata*, varying from 2C to 64C, were reported by Pal et al. (2004). Thus, the differences, which were about 200% between the values found for *Myrsine* species in this study and the value observed for *Myrsine africana*, can be related to the C value of *Vigna radiata* used as reference.

Values close to *Myrsine umbellata* and *Myrsine coriacea* species were reported for *Cyclamen purpurascens* Mill. (2C = 6.60 pg) and *Dodecatheon meadia* L. (2C = 5.58 pg). Higher DNA contents were described for *Cyclamen coum* Mill. (2C = 13.56 pg), *Soldanella pusilla* Baumg. (2C = 12.36 pg), and lower values for *Soldanella hungarica* Simonk (2C = 3.16 pg) and *Primula vulgaris* Huds (2C = 0.47 pg) (Bennett and Leitch 2012). The interspecific variation for the 2C DNA value found in this study, as for other species of Primulaceae (Bennett and Leitch 2012), suggests the occurrence of karyotype changes.

As well as for FCM, karyotype data about *Myrsine* species in the literature are very limited, with only the chromosome number reported (Beuzenberg and Hair 1983, Dawson 1995, Dawson 2000, Molero et al. 2002, Molero et al. 2006, Rice et al. 2015). In vitro *Myrsine* plantlets were fundamental for providing sufficient quantities of roots for the cytogenetic study independent of the reproductive period. Meticulous standardization of the antimitotic agent and enzymatic maceration were also essential for accurate chromosomal characterization.

Chromosome number 2n = 46 (Beuzenberg and Hair 1983, Dawson 1995, Dawson 2000, Molero et al. 2002, Rice et al. 2015, present study) and 2n = 48 (Molero et al. 2006) had been reported, but this was the first record of 2n = 45. The odd chromosome number 2n = 45 was well-marked by absence of the homologue pair of the chromosome 23 (Fig. 2). So, other cytogenetic approaches should be performed from *Myrsine* individuals separately to know the cause of this aneuploidy.

Some chromosome groups determined by statistical analysis are morphologically distinct, such as chromosomes 22 and 23 of *Myrsine coriacea*. Although clustered (Fig. 3b), these chromosomes are cytogenetically distinct, with 22 being acrocentric and 23 submetacentric (Fig. 2b, Table 2). Likewise, distinct chromosomes clustered in *Myrsine parvifolia* (Fig. 3a, Table 2) and *Myrsine umbellata* (Fig. 3c, Table 2). Chromosome 1 of *Myrsine coriacea* and 1 and 2 of *Myrsine parvifolia* and *Myrsine umbellata* presented the highest contrast, considering the morphology and Euclidean distances (Fig. 2, Fig. 3). Similarities and differences regarding relative size (% size in relation to sum of the mean values of total length, Table 1) were shown between *Myrsine coriacea* and *Myrsine umbellata* through the Scott-Knott test. The similarities, which were shown for some chromosomes, imply that these species could have originated from a common ancestor. The distinct chromosomes are likely to be attributed to karyotype changes that happened throughout their evolution, altering the chromosome relative size and contributing to taxa diversification. Comparative investigations of the karyotypes of related species have usually been applied to infer the evolutionary role of karyotypic modifications in different taxa and to describe the pattern and directions of chromosomal evolution within a group (Stebbins 1971, Soltis and Soltis 2012, Amaral-Silva et al. 2016).

Based on the constant chromosome number displayed by *Myrsine* species, interspecific variation of the nuclear 2C value between *Myrsine parvifolia* compared to *Myrsine coriacea* and *Myrsine umbellata* was also caused by karyotype alterations. The changes to the nuclear DNA content have also been attributed to structural rearrangements and/or heterochromatin polymorphisms (Pellicer et al. 2014, Amaral-Silva et al. 2016).

In conclusion, the first karyotype description and data about nuclear 2C value were shown for three *Myrsine* species. Besides of the comparison between them, these data represent the basis to understand karyotype evolution in *Myrsine*.

### Author contribution statement

The authors Carvalho RF, Amaral-Silva PM, Spadeto MS and Clarindo WR conceived, designed and conducted the tissue culture, flow cytometry and cytogenetic approaches. Carvalho CR contributed the flow cytometry analysis. Amaral-Silva PP and Carrijo TT collected and identified the *Myrsine* species. Nunes ACP did the statistical analysis. All authors contributed equally to manuscript editing and revision and approved the final manuscript for submission.

### Conflict of interest

The authors declare they have no conflict of interest.
